# Practical considerations for handling implants in proton therapy

**DOI:** 10.1002/acm2.70322

**Published:** 2025-10-30

**Authors:** Gloria Vilches‐Freixas, Vicki Trier Taasti, Ilaria Rinaldi, Mirko Unipan

**Affiliations:** ^1^ Department of Radiation Oncology (Maastro), GROW Research Institute for Oncology and Reproduction Maastricht University Medical Centre+ Maastricht The Netherlands; ^2^ Danish Centre for Particle Therapy Aarhus University Hospital Aarhus Denmark

**Keywords:** clinical instructions, CT image artifacts, metal implants, proton therapy

## Abstract

**Background:**

CT simulation scan is typically used for proton therapy planning. However, non‐tissue‐like materials are often not correctly characterized by the CT conversion curve, and medical implants of high‐Z materials, such as metals, can introduce artifacts in CT images. These can negatively affect the accuracy of dose calculations, ultimately impacting both treatment planning and dose delivery.

**Purpose:**

To present our clinical procedures for managing implants in patients treated with proton therapy.

**Methods:**

We categorized implants into two groups—‘small’ and ‘large’—based on their size relative to the CT voxel and their effect on image quality. To assess the influence of different implants on dose calculations, we conducted dose evaluations in a few patients with different implants. Our key considerations included the selection of appropriate CT window‐level settings for implant contouring, the necessity of performing material overrides weighing the tradeoff between the time spent on contouring and the impact on the dose distribution, as well as artifact mitigation strategies. The proposed procedures were derived from these dose evaluations and our clinical experience.

**Results:**

For small implants, made of titanium or tantalum, we can rely on our CT conversion curves, without requiring material overrides. Conversely, larger implants, such as silicone breast implants, metal hip prostheses, or air pockets, require material overrides to ensure an accurate representation of stopping‐power ratios and to minimize dose discrepancies. The general rule is to avoid proton beams passing through metal implants when possible.

**Conclusions:**

We have presented a comprehensive and standardized strategy for managing implants in our routine clinical practice. These strategies have been specifically developed for our clinical setup based on pragmatic considerations and may not be universally applicable. However, we believe they can offer valuable insights for other proton therapy centers facing similar challenges and looking to enhance their protocols for implant management.

## 1 INTRODUCTION

Proton therapy planning typically relies on a computed tomography (CT) scan of the patient.^1^ To estimate the proton stopping‐power ratio (SPR), needed for proton dose calculation, a CT conversion curve is used.^2^ For patients who have undergone mastectomy, tissue expanders or breast implants are often placed before radiotherapy.^3^, ^4^, ^5^ However, non‐human materials, like silicone used in breast implants are not correctly handled by a CT conversion curve,^6^, ^7^ which can cause inaccuracy in the proton dose calculation.

High‐Z materials, such as metal surgical implants, can cause artifacts in the CT image due to photon starvation and scattering. These artifacts are often seen for metallic dental fillings and surgical clips (frequently seen in post‐surgery radiotherapy),^8^, ^9^, ^10^ as well as for metal ports of tissue expanders.^11^ Such artefacts can both reduce the dose calculation accuracy and hinder delineation. Moreover, the metal object often looks larger than its actual dimension, and the visual appearance of the metal object is very dependent on the chosen window‐level setting.^12^

Given the large number of patients with implanted objects that degrade CT image quality, several strategies have been developed to address metal artifacts.^13^ All major CT vendors provide reconstruction algorithms for metal artifact reduction (MAR).^14^ However, even with MAR, some artifacts may persist. Moreover, metal objects may not have a CT number that correctly represents the photon attenuation of the metal it theoretically should cause. For example, in 12‐bit CT images, the maximum CT number value is 3071 HU, which is far below the actual CT number for implants like titanium.^15^ Newer 16‐bit CT scanners improve upon this limitation,^16^, ^17^ though small metal objects still suffer from partial volume effects that lead to inaccurate CT numbers. To address this, metal objects and surrounding artifacts are often segmented and overridden with the expected CT numbers. However, the exact knowledge of the correct CT number (or SPR) to use for the override is hard to achieve, and the size of the object to override is also hard to judge.^13^

Metal artifacts, particularly when located near the target area, can undermine the accuracy of radiotherapy, affecting both dose calculation precision and the delineation of the clinical target volume (CTV). Strategies to mitigate these artifacts are essential, and Rousselle *et al*. have provided a comprehensive review of methods aimed at minimizing metal artifacts within the CTV.^18^ In some cases, proton centers may choose to increase proton range uncertainty to compensate for these artifacts.^19^ However, this approach could inadvertently increase the dose to organs‐at‐risk (OARs). Therefore, exploring more effective mitigation strategies is preferred rather than simply increasing the range uncertainty.

This document aims to describe how implants are handled in the proton treatment planning process at our clinic, to establish a standardized approach for all clinical proton applications. Implants considered in this manuscript include clips, coils, breast implants, pacemakers, wires, dental fillings, air, and feeding tubes. The considerations presented here are tailored to our specific clinical context, including CT scanner settings, treatment planning system, treatment procedures, patient population, and patient workflow. This document will detail the strategies used at our proton clinic to manage implants in CT images, outlining specific approaches for each implant type, supported by examples from our patient cases. While these strategies are specifically developed for our clinical setup and may not be universally applicable, we believe they can offer valuable insights to other clinics looking to enhance their protocols regarding implant management.

## 2 MATERIALS AND METHODS

### 2.1 Technical equipment

At our clinic, we have two CT scanners, Somatom Confidence and Drive (Siemens Healthineers, Forchheim, Germany). All CT images are reconstructed with iterative reconstruction (SAFIRE or ADMIRE, strength 3) and a quantitative kernel (Qr40) with beam hardening correction (iBHC) for bone. All scans are 12‐bit images. The slice thickness depends on the treatment site (range: 1–3 mm). For most patients, a field‐of‐view of 500 mm is used, resulting in a voxel size of 0.98 mm in the axial plane. For brain cancer patients, the field‐of‐view is always 330 mm, and for patients who do not fit within the field‐of‐view of 500 mm, an extended field‐of‐view of 650 mm is used. Iterative metal artifact reduction (iMAR) is always applied for brain and breast cancer patients, while it is used only if needed for other patients. The iMAR settings used for each patient group are listed in Table 1.

**TABLE 1:** iMAR settings for each anatomical region or indication. Abbreviations: ICD—Implantable cardioverter defibrillator.

MAR option (abbreviation)	Implants
Dental fillings (de)	Dental fillings
Extremity implants (ex)	Knee and elbow prosthesis
Hip implants (hi)	Hip prosthesis
Neuro coils (ne)	Neuro implants, clips breast, clips liver
Pacemaker (pa)	ICD, pacemaker, tissue expander
Shoulder implants (sh)	Shoulder prosthesis
Spine Implants (sp)	Screws, metal plate fixations of the spine
Thoracic coils (th)	Sternum wires

Most of our proton therapy patients are scanned with single‐energy CT (SECT) with a fixed tube voltage of 120 kVp, and a CT conversion curve is used to convert CT numbers to SPRs, created based on current guidelines.^2^ Brain cancer patients are scanned with dual‐energy CT (DECT),^20^ and the Siemens DirectSPR algorithm is used for proton dose calculation. To account for titanium in the dose calculation, based on both SECT and DECT, a horizontal line with a constant SPR corresponding to titanium (SPR = 3.1 at 100 MeV) is introduced in the CT conversion curves (see Figure S1 in the supplementary material).

Proton treatment plans are created in RayStation 12A (RaySearch Laboratories, Stockholm, Sweden). All plans are robustly optimized and robustly evaluated (range uncertainty of 3% for SECT and 2% for DECT, and setup uncertainties from 1–5 mm depending on the anatomical site),^21^ and Monte Carlo dose calculation with 1% uncertainty is used for both plan optimization and evaluation.

Our Mevion S250i Hyperscan proton system^22^ is equipped with orthogonal kV imaging and the Imaging Ring cone‐beam CT (CBCT) system (medPhoton, Salzburg, Austria). For patients with implants, daily CBCT imaging is performed to ensure the correct positioning during the whole treatment course. Most patients in our proton clinic receive a weekly repeated CT.

### 2.2 Types of implants

At our proton clinic, implants are categorized into two subgroups: *small* and *large* implants (Table 2). The category depends on the implant size compared to the CT voxel size (with small implants having a similar size as the CT voxel) and the severity of the artifacts the implant creates in the image. Generally, for each implant seen in a patient CT scan, an effort is made to find out which material the implant consists of by contacting the surgeon. For brain cancer patients, there is a general agreement across the region (Limburg, The Netherlands) only to use titanium implants.

**TABLE 2:** Types of implants we see in patients, classified according to their typical size compared to the CT voxel size.

Small	Large
Neuro coils	Breast tissue expanders (with valve)
Neuro meshes	Silicone implants (rubber shell thickness < 1 mm)
Neuro screws	Saline implants (rubber shell thickness < 1 mm)
CT markers (radiopaque lead balls)	Dental fillings
CT wires used for breast patients	Portacaths
Titanium clips	Pacemakers
Tantalum clips	Pacemaker wires
Titanium suture staples	(Coronary) stents
Coated feeding tubes	Air in the target volume
	Metal hip, shoulder and spine implants
	Gastric bands

### 2.3 Dose distribution evaluations

To evaluate the impact of the window‐level settings on the resulting contour size, we had the metal implant in a few patients contoured using different window‐level settings to assess the contour size and the effect on the dose distribution.

In the case of small implants, we also assessed the impact of overriding the implant with its specified material compared to not overriding. For the latter, the dose calculation relies solely on the conversion of the CT numbers in the voxels occupied by the small implant to the corresponding SPRs. The CT scan was duplicated, and the dose was recomputed on the CT scan without material override.

In some situations, like air in the target volume, we also performed a robust evaluation of the treatment plan on the CT without material override.

In case the material of the implant is not known, it is an alloy, or it is not available in the material list in Raystation, our approach to quantify (get a feeling of) the uncertainties is to duplicate the CT and structure set twice, contour the implant, and potentially also contour the artifacts. The implant is then overridden to the two materials in the Raystation material list which are closest to the actual (or assumed) implant material, one material override on each of the two copies of the CT. For metal implants, the two evaluated override materials are often titanium and steel.

In case there are imaging artifacts inside the CTV, or the proton beam goes through the artifacts, we override the artifacts to the surrounding materials, for example, to muscle if the artifacts are in the heart or muscle, or to adipose if the artifacts are in the breast. The dose is then robustly recomputed on the duplicated CTs with the material overrides. For these evaluations, we set the robustness parameters to 3% (SECT) or 2% (DECT) range uncertainty and 1 mm setup uncertainty (or 2 mm in case of esophagus patients following the PROTECT trial protocol^23^) as used for repeat CT evaluation. Comparing the different dose evaluations, the influence of the material overrides can be assessed.

## 3 RESULTS

The clinical procedures for how to handle each implant is given in the supplementary material. There, all implants listed in Table 2 are described separately. Here we only describe the evaluations we have performed to establish the considerations presented in the supplementary material, therefore not all individual implants are mentioned here.

### 3.1 Small implants

#### 3.1.1 Titanium clips

**FIGURE 1:**
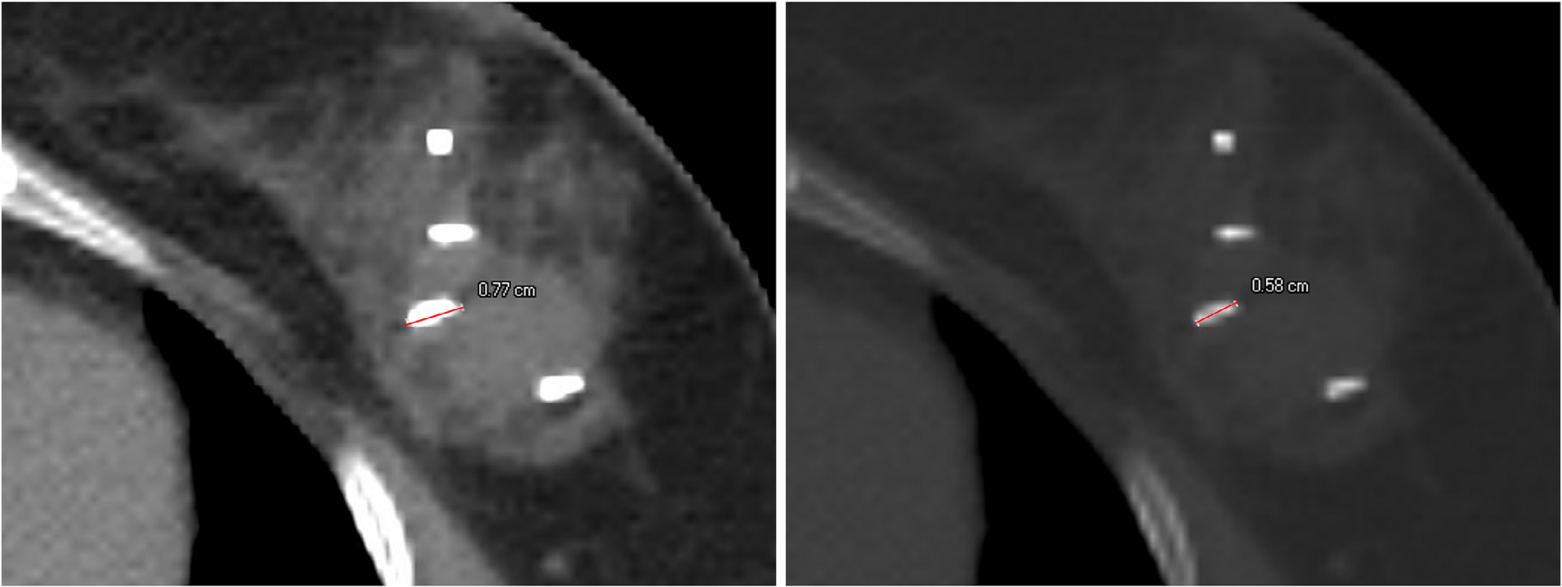
Example of the importance of choosing the correct window‐level setting when assessing the size of clips. The actual size of the clips in this patient is 1.5 mm in diameter and 5 mm length, known from manufacturer specifications. (Left) Medistanium window‐level setting (40/400 HU; the size of the clip is overestimated in this window‐level setting; measured length of the clip is 0.77 cm). (Right) Bone window‐level setting (450/1600 HU; here the size of the clips is closer to reality; measured length 0.58 cm).

**FIGURE 2:**
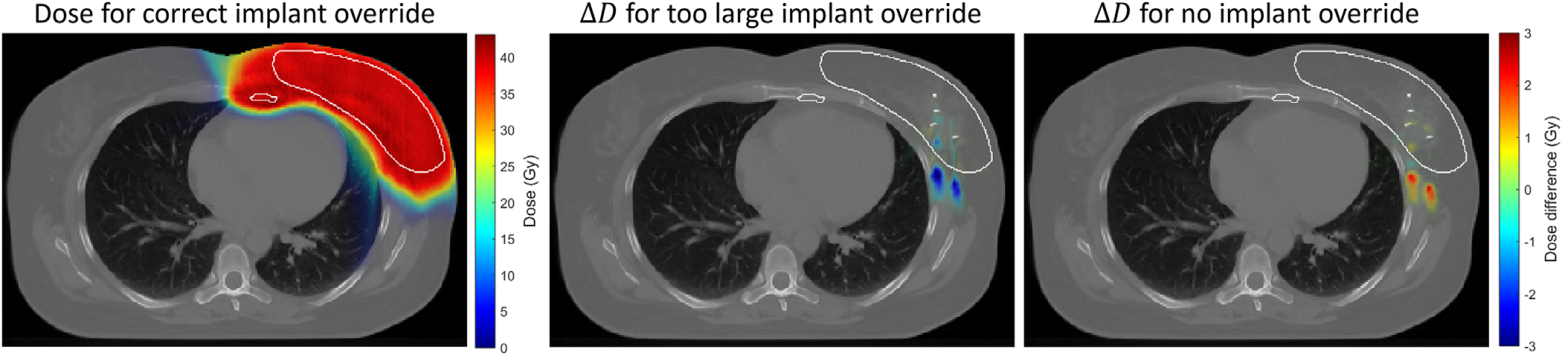
Effect of implant override on the dose distribution—based on the same patient as shown in Figure 1. At the left, the dose distribution on the CT image with the correct implant contour is shown. At the middle and at the right, the dose differences are shown for the CT with a too large contour override (middle) and the CT with no contour override (right). The dose difference is calculated as Δ D = D_toolarge/nooverride_—D_correctovveride_. The white contour shows the CTV. The titanium clips are visible within the contour in the middle and right image (four titanium clips are visible in this CT slice). Dose differences are seen downstream of the clips in both the middle and right images, but with different sign. Also note, the dose differences are generally larger in the middle image for the CT with the too large contour override.

Figure 1 illustrates the importance of choosing the right window‐level setting for assessing the size of the clips. In the **Mediastinum** (40/400 HU) setting, the size of the clip is overestimated, while the size of the clip in the **Bone** (450/1600 HU) setting is closer to the true size of the clip. Figure 2 evaluates the impact of performing a material override of the clip, with a delineated structure which is too large, in comparison to not overriding. Three scenarios were assessed: (1) accurate clip size delineation with titanium override; (2) overestimated clip size with titanium override; (3) no override. In scenario 3, the SPR of the implant would be estimated based on the conversion curve which would be affected by the partial volume effect, lowering the CT numbers and thereby the estimated SPR. The 2D gamma map (not shown) overlapped very well with the shown dose differences and gave a gamma passing rate of 97.7% for scenario 2 and 99.4% for scenario 3, for a gamma criterion of 1%/1 mm and a dose cut‐off corresponding to 20% of the prescription dose. The dose differences for scenarios 2 and 3 are seen in similar position, downstream of the clips (anterior oblique fields), but in opposite directions compared to scenario 1. Overestimating the clip size led to under‐dosage downstream the clips, while not overriding caused over‐dosage but slightly lower. The dose differences in the OARs were negligible. To assess the accuracy of these TPS dose comparison we also recalculated the doses with an independent Monte Carlo dose calculation, FRED,^24^ which we use for routine log‐file‐based patient quality assurance. The TPS and FRED handle material overrides differently, but similar trends were obtained with both, whereby we concluded that the trend seen could be trusted.

It should be noted that we in general do not know the correct size of the implant, thus scenario 1 can be seen as an ideal scenario only achievable for a few patients where the implant specifications are known. Thus, to be conservative, it is preferred not to delineate the clips, as overestimating the dose outside the target is better than underestimating it, which could lead to an underestimation of the dose to the OARs (heart and lungs). When not overriding, the SPR is estimated by the CT conversion curve which includes titanium (see Figure S1 in the supplementary material). For small implants, like titanium clips, the partial volume effect will reduce the CT number whereby the estimated SPR to be lower than the one for titanium, which should also be the case if the full CT voxel is not occupied by titanium.

#### 3.1.2 Tantalum clips

For patients with tantalum clips, typically with a diameter of ∼5 mm, iMAR reconstruction is required to minimize surrounding image artifacts. The consequence of not overriding the clips and the artifacts or wrongly overriding them was evaluated in the same way as for titanium clips. When tantalum clips were delineated too large, the dose distribution showed localized under‐dosage downstream of the clips (5%–6%), while an over‐dosage (5%) was seen for no override. The impact on OARs was negligible. Tantalum clips showed a larger impact on dose distribution compared to titanium due to the higher atomic number (Z = 73 vs. Z = 22), with gamma passing rates for 1 mm/1% of 89% for too large override and 87% for no override, respectively.

**FIGURE 3:**
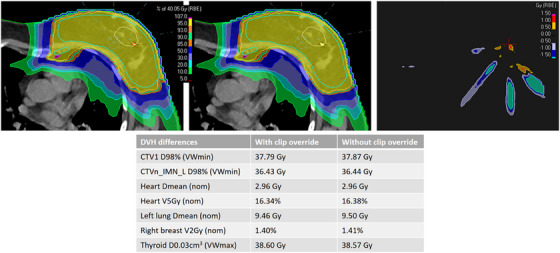
Example of the effect of not overriding tantalum clips on the dose distribution. (Top left) The plan is optimized with a tantalum override of the clip (red contour in the middle of the breast; mass density 16.69 g/cm^3^). (Top middle) The plan is recalculated with no override of the clip. (Top right) Dose difference (dose with override minus dose without override); here the red cross marks the position of the clip. (Bottom) Dose‐volume histogram parameters from the two dose distributitions. nom—nominal dose distribution; VWmin—voxel‐wise minimum dose distribution; VWmax—voxel‐wise maximum dose distribution (see references 21, 25).

In addition to the gamma pass rate analysis, a proton plan where the clips were overridden to tantalum (with the correct clip size) was compared to a plan where no clips were overridden (Figure 3). Dose evaluations showed only small dose differences between the proton plans with and without tantalum clip overrides. Differences were mostly observed downstream of the CTV (2%–4% dose variation), mainly affecting the low‐dose areas outside the target volume, while the effect on the dose‐volume histogram parameters were negligible. Therefore, it was concluded that not overriding the tantalum clips is acceptable for a conservative approach.

#### 3.1.3 Coated feeding tubes

Esophagus cancer patients often require tube feeding,^26^ and sometimes the tube is coated in a thin layer of iodine which enhances the CT numbers. We conducted a study to evaluate the impact of feeding tubes on the proton dose distribution. We tested two approaches: one where the tube was overridden to water (approach 1) and another where no override was applied (approach 2). Proton plans for each approach were optimized with a setup uncertainty of 5 mm and range uncertainty of 3%, as used clinically for plan optimization. Each optimized plan was recalculated for the other scenario. The results showed that the dose differences between the two approaches were minor, around 1% overall, with some voxels experiencing up to 3% (approach 2) or 5.7% (approach 1) dose differences. As the two optimized plans did not markedly differ, we found that the override did not have clinical impact, and therefore, we decided not to override the feeding tube to water to ease the workflow. Robust optimization with 3% range uncertainty was deemed sufficient to ensure plan reliability. We argued that the differences in the two evaluated scenarios were more extreme than, for example, situations where the feeding tube might shift a little within the CTV, so the conclusions also apply to feeding tube shifts within the esophagus during treatment.

### 3.2 Large implants

#### 3.2.1 Silicone breast implant

The SPR of silicone is not correctly estimated using a SECT‐based conversion curve due to the relative high atomic number of silicon (Z = 14) which increases the CT number of silicone, while the mass density is below that of water.^6^ Due to the large size of silicone breast implants, a material override is therefore needed to be able to calculate proton dose accurately. However, the override materials in RayStation 12A do not include silicone (plastic material, consisting of H, C, O, and Si). Alternatives are to override the implant contour to either water or silicon (pure material, Z = 14) and then adjust the mass density to properly match the proton range. We have investigated which of these two override strategies was preferable (see details in the supplementary material S3B.1b). We concluded that overriding to a material composed of water but with a mass density 0.934 g/cm^3^, corresponding the SPR measured by Michalak *et al*.,^6^ was preferable, while to override to silicon is not clinically feasible because the mass density for the override will be energy dependent (see supplementary material section S3B.1b).

#### 3.2.2 Pacemakers wires

**FIGURE 4:**
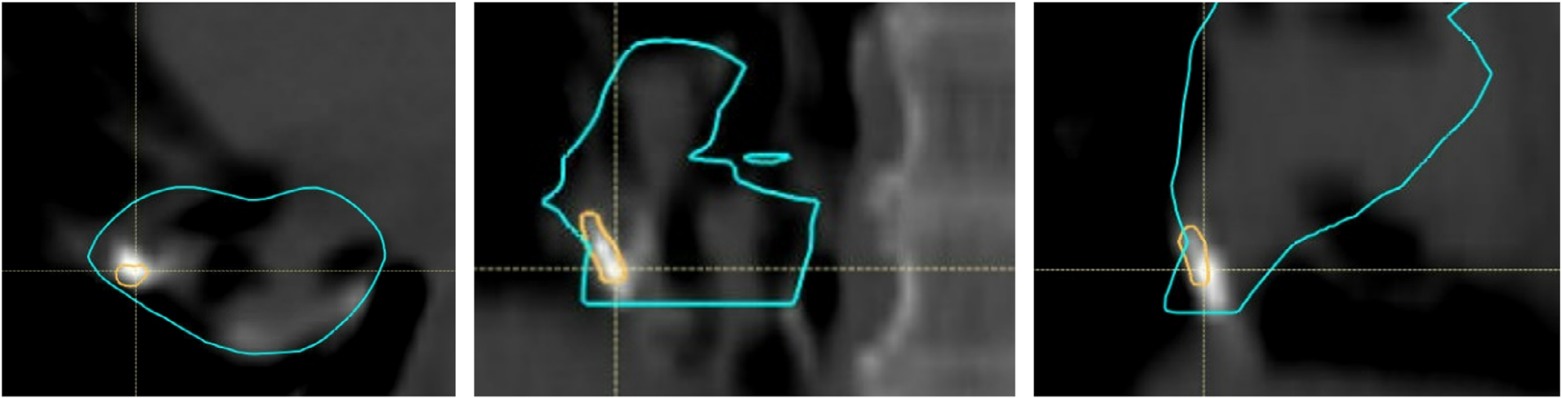
Example of motion of an implant. The implant was delineated on the CT50ex phase and the structure was copied to the average CT. However, this delineation did not accurately represent the structure on the average CT. High‐intensity voxels partly fall outside the orange contour, while not all voxels inside the orange contour are high‐intensity. This would lead to an incorrect override of some voxels inside the target volume (blue contour). Therefore, the implant has to be contoured directly on the average CT for accurate plan optimization. Window‐level setting Bone (450/1600 HU).

Thoracic tumor patients are typically treated in free‐breathing at our clinic, and the treatment plan is based on a 4DCT.^27^, ^28^ The delineations are made on the 50% expiration phase (CT50ex), while the plan is created on the average CT (CTavg). We evaluated the effect of delineating pacemaker wires on CT50ex and rigidly transfer the contour to CTavg. We found that this could result in a slightly wrong contour and thereby a wrong override area (Figure 4). It was therefore decided to delineate implants directly on the CTavg.

#### 3.2.3 Air pockets in the esophagus

**FIGURE 5:**
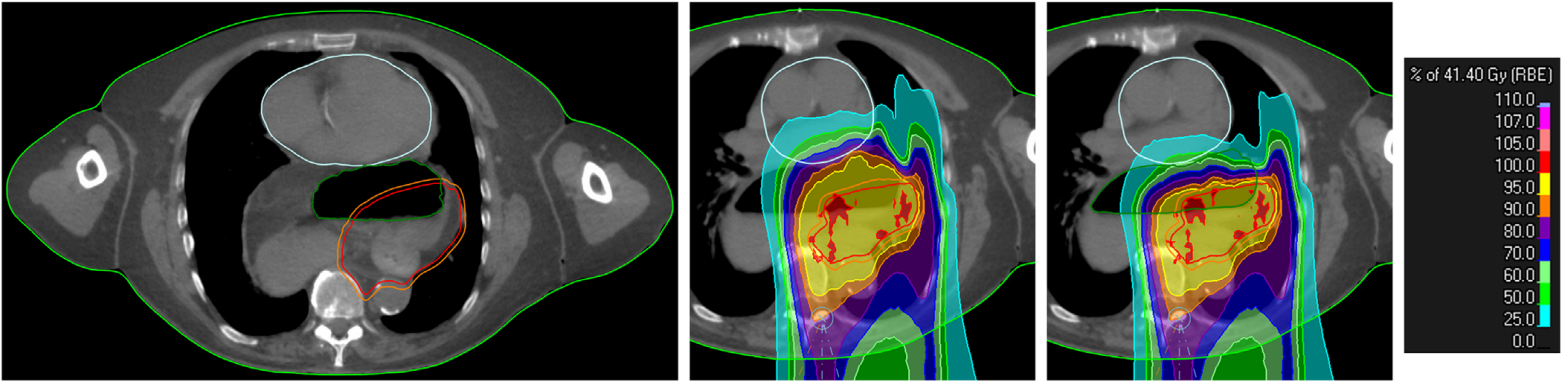
Example of esophagus cancer patient with air (dark green contour) in the target (the red contour is the internal target volume (ITV), and the orange contour is the ITV expanded by 3 mm, this is the target volume). In the middle figure, the proton plan optimized on the original CT (no override of the air) is seen—the shown dose distribution is the voxel‐wise minimum (VWmin) dose distribution, which is used to evaluate the target coverage. The right figure shows the VWmin for the robust re‐evaluation of the plan optimized on the CT without air override but evaluated on the CT with an override of air to water with a density of 1 g/cm^3^. The dose in the target is similar, but the beams shoot less into the heart (light blue contour) on the CT with the override (right).

Air cavities in the gastrointestinal tract can have large impact on the dose distribution due to the low density of air, causing a proton beam over‐shoot. We studied the impact of air in the target volume using planning CTs from six esophagus cancer patients. We contoured the air volume and created two CT copies, overriding the air contour with densities of 0.5 g/cm^3^ and 1.0 g/cm^3^ to simulate partial and full disappearance of the air. Proton plans consisting of three posterior beams (left‐posterior oblique, anterior‐posterior, and right‐posterior oblique) were optimized on each of the three CTs and robustly evaluated on the two other CTs (one comparison shown in Figure 5). The dose‐volume histograms showed no relevant differences (not shown), indicating that robust optimization alone was sufficient.

To assess the effect of air changes during treatment, we simulated air cavities by drawing cylinders of varying diameters (5 to 25 mm) for two patients. We found that air volume changes larger than 20 mm influenced the mean heart dose by more than 1.2 Gy (prescription dose of 41.4 Gy; see Figure S18 in the supplementary material) but had minimal impact on the target dose. Based on these results and our clinical experience, we do not contour air pockets smaller than 15 mm. For air pockets larger than 15 mm, we contour and override them to water (density adjusted to 0.5 g/cm^3^), then evaluate the plan on a CT copy with no override. Due to the posterior beam directions, we typically use for esophagus cancer patients, we have not evaluated air pockets in OARs like the stomach or trachea.^27^

## 4 DISCUSSION

We have presented the dose evaluations we conducted to define our clinic's implant management strategy. The extensive list of evaluated implants and our approach for their handling within our clinical practice are detailed in the Supplementary material. In general, our clinical strategy is not to override small implants, but always to override large implants (except saline implants). This is the reason we divided the implants into these two categories.

Our findings indicate that the window‐level setting impacts the precision of implant delineation, which affects the dose distribution in the final treatment plan if the contour has a material override. In general, the bone window‐level setting is a good approach for delineating metal implants (or any high attenuating objects), independent of TPS and CT parameters. As seen from Figure 1, small metal implants can cause blooming artefacts, which make the implant seem larger than it is, but this can to some extend be counteracted by using the bone window‐level setting. If a small implant is contoured too large, it can lead to under‐estimating the actual dose to normal tissue resulting in over‐dosage. We compared the impact of not delineating small implants, and relying solely on the CT conversion curve, to delineating a too large area and thus overriding a too large volume, as a too large contour could be the result of delineation using a wrong window‐level setting. It is important to note that human contouring variability can affect the delineation of small implants or clips even with the optimal window‐level setting.^29^ For example, the brush size used for delineation can influence the final size. After conducting dose calculation checks in RayStation, we decided against overriding the small implants. It is crucial to find a tradeoff between contouring time and the impact on the dose distribution. In general, small titanium or tantalum implants can be handled by relying on the CT conversion curve for proton dose calculation, because our CT conversion curves include a horizontal segment at high CT numbers with a constant SPR corresponding to titanium. It should though be noted that the partial volume may reduce the CT number of the implant compared to the theoretical CT number of the implant material.^30^ Nevertheless, it is advised to avoid proton beams passing through these implants when possible. In some cases, implants might still need to be contoured since this contour is used during daily imaging to evaluate the match, but no override for dose calculation purposes is needed.

For larger implants, it is essential to contour and override them to the appropriate material. For large implants, the blooming artefact, which can make the implant look larger, is relatively a smaller effect compared to the actual size of the implant. Moreover, for non‐metallic implants, like breast implants, blooming is less/not prevalent. The contouring accuracy can therefore be relatively higher than for small implants. On the other hand, overriding the implant is more important. Even titanium implants, which are included in the CT conversion curve, require overriding due to potential CT number non‐uniformity within the material. When possible, proton beams through the implant should be avoided, and surrounding streak artifacts should be carefully evaluated to determine the reliability of the CT numbers in the affected areas.

Numerous studies have examined the effect of implants on CT image quality for contouring and their impact on photon and proton dose distribution.^13^, ^31^, ^32^, ^33^, ^34^, ^35^, ^36^ Most centers currently use MAR algorithms.^37^ Additionally, DECT has been proposed to minimize metal artifacts by generating a virtual monoenergetic image (VMI) at higher energy levels, and combining MAR algorithms with VMI has proven to be the most effective method.^38^, ^39^ Currently, most DECT acquisition methods only work for static anatomical regions,^40^ but dual‐layer DECT and photon‐counting CT will be able to provide DECT acquisition for the full body.^41^, ^42^ Some modern CBCT systems also include MAR capabilities.^43^

It should be noted that this document has primarily examined the effect of metals on the CT images, but it did not assess the effects of metal on the proton beam. Protons scatter when they pass through metal, which can affect the dose distribution.^44^ This ought to be accounted for when applying Monte Carlo dose calculation, but more importantly, we generally try to avoid having proton beams pass through metal implants to prevent the metal from affecting the dose distribution.

The strategies outlined here are based on our clinic's experiences and available equipment, and several of the presented considerations are of pragmatic nature. While these strategies may not apply to all centers, similar considerations may be relevant to others. Currently, there are no standardized guidelines for handling implants, as practices can vary significantly depending on the equipment and experience of each center. Therefore, we believe sharing our strategy can be useful for others. Our descriptions are limited to the implants we have encountered in our patients, and other implant types may require different management strategies. We continue to collect examples as new implant materials are identified in our patients, as we acknowledge that the considerations stated here could be case‐specific, thereby expanding our clinical protocol for handling implants.

## 5 CONCLUSION

We have described our clinical strategies for handling different implants seen in cancer patients treated with proton therapy, along with the evaluations we have performed to guide our decisions when setting up our clinical procedures.

## AUTHOR CONTRIBUTIONS

**Gloria Vilches‐Freixas**: Conceptualization; methodology; formal analysis; investigation; writing—original draft; writing—review & editing; visualization. **Vicki Trier Taasti**: Conceptualization; methodology; investigation; writing—original draft; writing—review & editing; visualization. **Ilaria Rinaldi**: Conceptualization; writing—review & editing. **Mirko Unipan**: Writing—Review & editing.

## CONFLICT OF INTEREST STATEMENT

The authors declare no conflicts of interest.

## Supporting information



Supporting Information
