# Supplementary material for: Practical considerations for handling implants in proton therapy
Source: J Appl Clin Med Phys. 2025 Oct 30;26(11):e70322. doi: 10.1002/acm2.70322 (PMC12575054; doi:10.1002/acm2.70322)
Supplement: Supplementary file 1 — Supporting Information [file ACM2-26-e70322-s001.pdf]

Supplemental material

Practical considerations for handling implants in proton therapy

S1 Purpose

In this document, the considerations on how to handle implants in the proton treatment planning process at our clinic are collected. The aim of collecting these considerations was to have a uniform procedure for all clinical proton indications. A short description is given for each implant on how we handle it in the treatment planning process. The list of implants includes all implants we have seen in our proton therapy patients. There is therefore no guarantee that the list is complete. Moreover, the procedures described are developed based on our clinical situation (i.e., our CT scanner settings, our treatment planning system, our treatment planning procedures, and our patient workflow), our patient population, and our equipment, and other considerations might be needed for other clinics.

The implants are divided in two categories, small and large implants. These categories are based on the implant size compared to the CT voxel size and the severity of the CT image artifacts the implant causes. The description of the evaluations we have performed to develop these procedures is given in the main text. In this supplementary document, we describe the clinical procedures, including window-level settings used for delineating the implants and the material override strategies.

S2 CT conversion curves

As described in the main text, for small implants often a material override is not performed, and instead the dose calculation relies solely on the conversion of the CT numbers in the voxels occupied by the small implant to the corresponding proton stopping-power ratios (SPRs). Therefore, we will briefly describe our CT conversion curves (see Figure S1). In the main text, our two CT scanners and CT protocols are described. We have four clinically applied CT conversion curves, three for single-energy CT (SECT) and one for dual-energy CT (DECT) combined with the Siemens DirectSPR algorithm (Siemens Healthineers, Forchheim, Germany).

The three SECT-based conversion curves are generated following a recent guide[1]. For head protocols, the two CT scanners give similar CT numbers, whereby a single curve is used for both CT scanners, while for thorax and pelvic CT protocols, the two CT scanners differ in CT numbers, especially in the high-density region, and therefore a specific curve is used for each CT scanner. The voxel values in the DirectSPR maps are scaled SPR values, and a linear conversion curve is used.

For 12-bit CT images, the CT number of titanium is saturated at the highest CT number of 3071 HU, unless the partial volume effect decreases the CT number. To account for titanium, which is often used as an implant material, a horizontal line with a constant SPR corresponding to titanium (SPR = 3.1 at 100 MeV) was introduced in the CT conversion curves. For the three SECT curves, this constant SPR segment starts at a CT number of 2950 HU. Not to affect the slope of the curve in the bone region, an intermediate line section was introduced at CT numbers from 2500 HU. In a similar way, a fixed line segment for titanium was introduced in the otherwise linear DECT curve. The steeper slope of this curve entails that the SPR of titanium is reached at a lower voxel value, whereby no intermediate segment is needed.

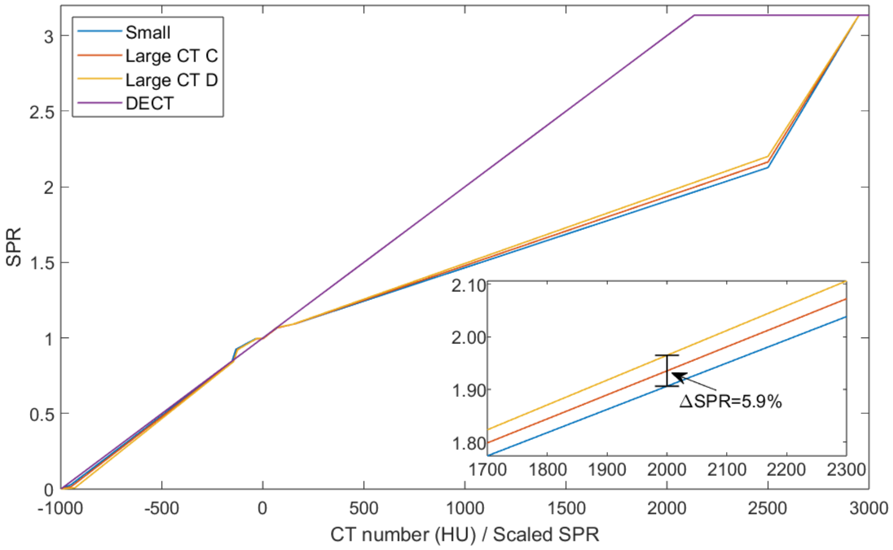

Figure S1: Conversion curves from CT numbers to stopping-power ratios (SPRs) for single-energy CT (SECT; blue, red, and yellow curves) and a de-scaling curve for dual-energy CT (DECT; purple curve). The blue curve ("Small") is used for head-and-neck cancer patients scanned at both CT scanners, while the red and the yellow curves are used for patients with tumors in the thoracic and abdomen region scanned at the Confidence ("C") and Drive ("D") CT scanner, respectively. The purple curve is for brain cancer patients scanned with DECT at both CT scanners. All curves have a flat segment in the high-density region with a fixed SPR value corresponding to the SPR of titanium at a proton energy of 100 MeV.

S3 Clinical considerations

S3A Small implants

For small implants, i.e. smaller or on the size of one CT voxel, we generally do not perform a material override. The specific considerations for each implant of this type are given below.

S3A.1 Titanium clips

Titanium clips are used for tumor localization after resection in head and neck (Figure S2) and breast cancer patients (Figure S3). As discussed in the main text (see Figure 1), the clips are best visualized with the **Bone** window-level setting (450/1600 HU), not to overestimate the size of the clips. Moreover, their small size (~1 mm for head and neck, ~2 mm for breast) compared to the CT slice thickness does not cause noteworthy imaging artifacts. In our clinic, we do not perform a material override for small titanium clips due to the negative impact on the dose distribution resulting from mistakenly contouring the implant too large, as seen in Figure 2 in the main text.

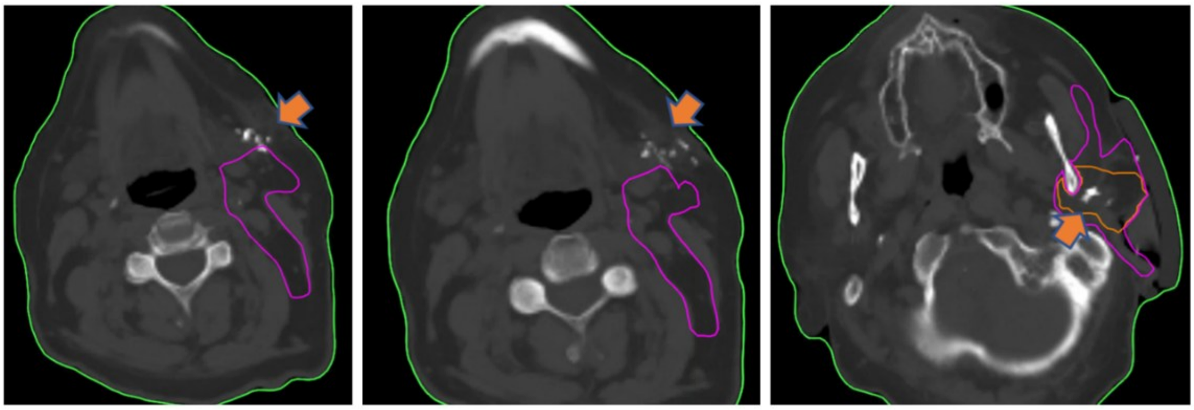

Figure S2: Example of CT scan of a head-and-neck patient with several titanium clips (marked with orange arrow) present immediately before and inside the elective clinical target volume (CTV1; purple) and boost CTV (CTV2; orange). Window-level setting Bone (450/1600 HU).

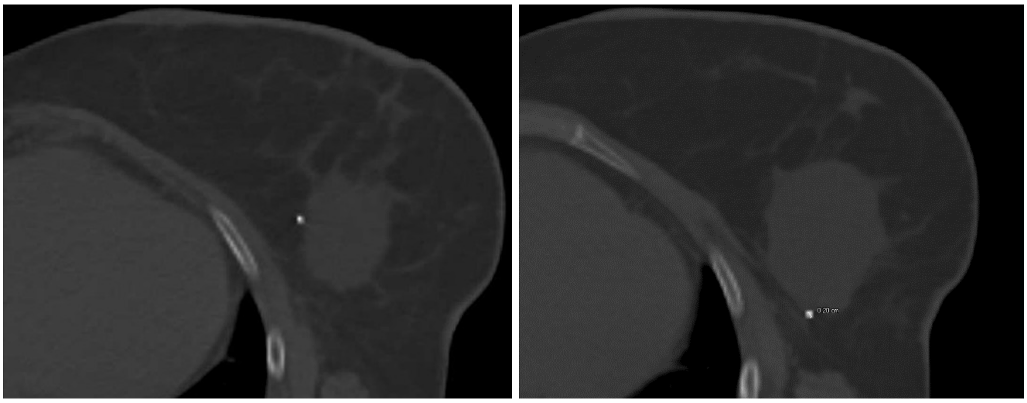

Figure S3: Example of CT scan of a breast patient with titanium clip, the two figures show two slices from the same patient. Window-level setting Bone (450/1600 HU).

S3A.2 Titanium suture staples

Surgical staples are composed of many tiny titanium clips[2]. On a CT image, this clip system looks like one clip of a somewhat larger size (~1 cm; Figure S4). Delineating this full region and overriding it to titanium will therefore overestimate the actual size. As for titanium clips (section S3A.1), no material override is used since the material is known to be titanium as it is included in the CT conversion curve.

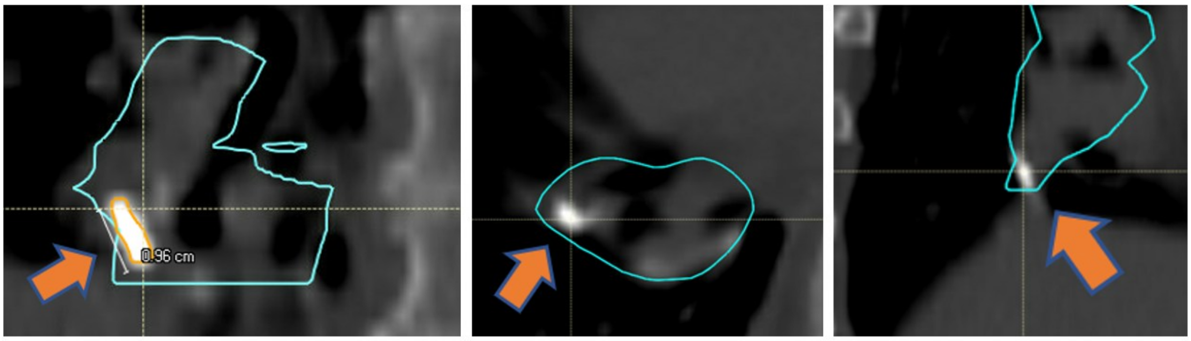

Figure S4: Example of a CT scan of a patient with titanium suture staples. On the CT images, the staples look like one large metal object, while in reality it is composed of many small titanium clips, meaning that the size is overestimated in the image. Coronal (left), transversal (middle), and sagittal (right) view of the same patient. Window-level setting Bone (450/1600 HU).

S3A.3 Tantalum clips

Figure S5 shows a patient with tantalum clips using window-level setting **Bone** (450/1600 HU). The same conclusions were found for the consequence of delineating tantalum clips too large as were found for titanium clips, the impact is just even higher (see Figure 3 in the main text). Therefore, for small tantalum clips, we also do not perform a material override and solely rely on the CT conversion curve, despite the curve only handling titanium and not tantalum. However, the incorrect results of this were found to be minor due to the small size of the clips. However, to ensure that the position of the clips remains the same during the treatment course, these clips are contoured with a margin of 5 mm (equal to the setup uncertainty), and their position is verified during daily imaging. To have a direct comparison of the impact of titanium and tantalum on the dose distribution, we used the patient seen in Figure 2 in the main text with titanium clips and overrode to tantalum instead. For the case of no override, this does not represent the true scenario. But due to the small size of the implant, the limitation of 12-bit CT images, and the plateau in over CT conversion curve (Figure S1), the dose in the no override case is similar for titanium and tantalum clips, whereby this simulated tantalum override is a good approximation. From Figure S6, it is seen that an override to tantalum has a larger influence on the dose distribution, as expected due to the higher atomic number of tantalum (Z=73 vs Z=22).

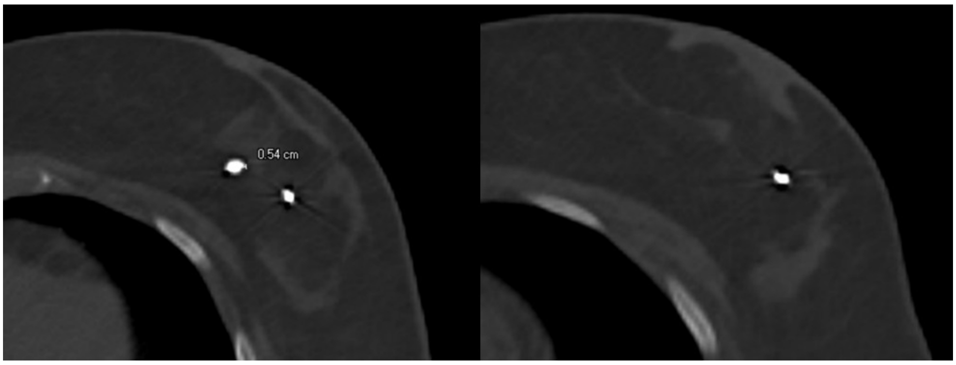

Figure S5: Example of CT scan of breast patient with tantalum clips, two different CT slices for the same patient. Remaining artifacts are seen as streaks around the clips despite applying iMAR reconstruction. Window-level setting Bone (450/1600 HU).

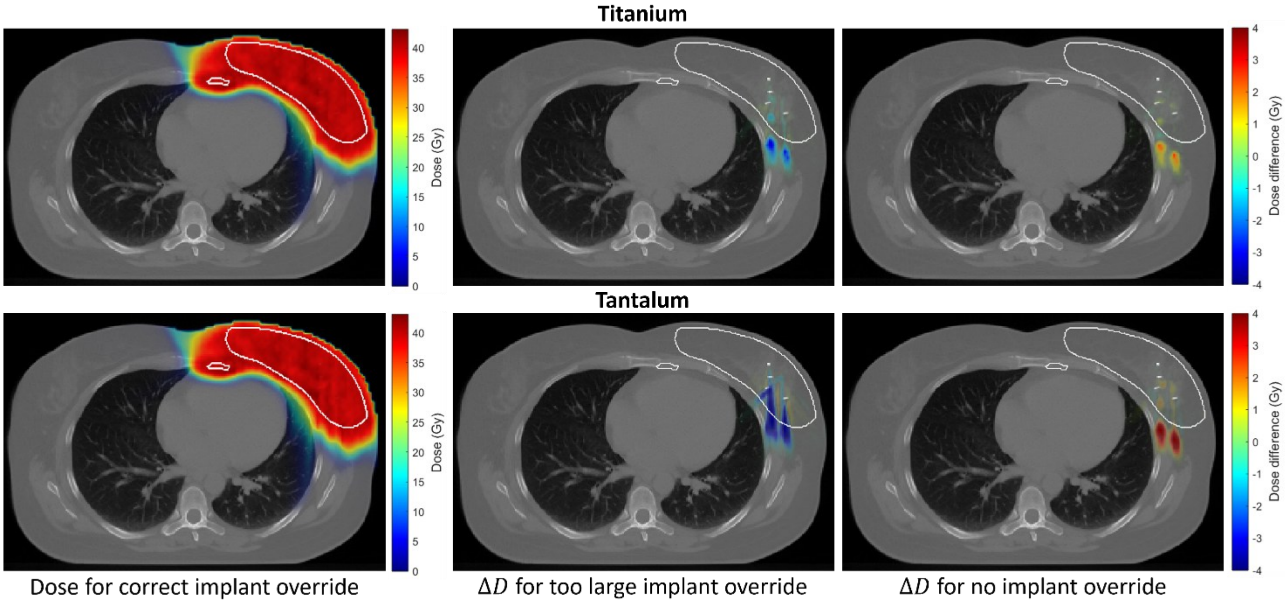

Figure S6: Dose comparison for titanium and tantalum override. The top row shows override to titanium (these subfigures are the same as seen in Figure 2 in the main text), while the bottom row shows override to tantalum (note, the actual implant is made of titanium, so the lower right subfigure is not a correct representation, but it is shown here for direct comparison of the effect of titanium and tantalum).

S3A.4 Neuro coils, meshes and screws

Clips used after tumor resection or coils for embolization often cause dense metallic artifacts. To address this, we always reconstruct CT scans of brain cancer patients using iMAR with the "neuro coil" setting (see all iMAR settings in Table 1 in the main text). Figure S7 shows a brain cancer patient with titanium screws in the skull with minimal artifacts around the small titanium implants, so no delineation and override are typically required, as the material is known to be titanium, and this is included in the CT conversion curve.

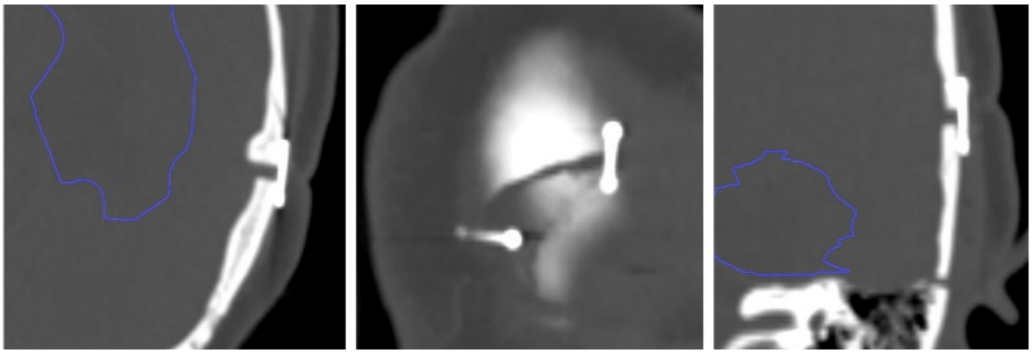

Figure S7: Example of CT scan of brain cancer patient with titanium screws (left: transversal, middle: sagital, right: coronal view). The blue contour is the primary gross tumor volume. The CT slice thickness is 1 mm. Window-level setting Dental (400/2000 HU).

S3A.4 CT markers

Radiopaque lead balls placed on the patient's skin during CT scans are absent during proton therapy and must be removed from the External contour before treatment planning. This is done by checking the External contour in the **Bone** (450/1600 HU) window-level setting (Figure S8). If it has been forgotten to remove the markers, we check the dose impact by duplicating the CT scan, removing the markers, and recomputing the dose. The resulting dose differences are typically minor, so if the markers are accidentally left in the External contour and the proton beams pass through them, the plan can typically still be accepted.

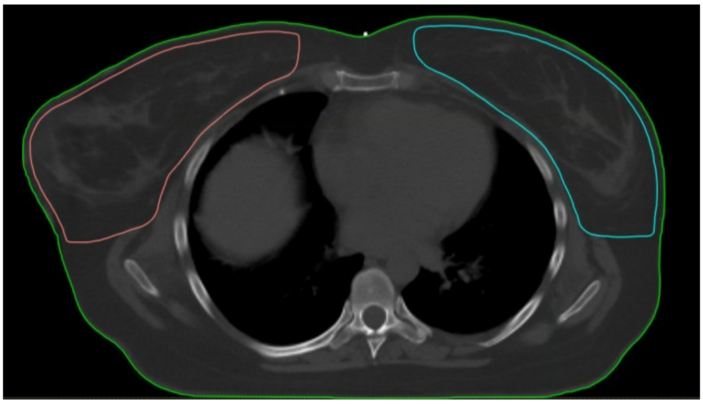

Figure S8: Example of CT scan of a breast patient with radiopaque lead ball placed on the sternum. Window-level setting Bone (450/1600 HU).

S3A.5 CT wires (breast patients)

Lead wires placed around the palpable breast help reduce variation in delineating the breast target volume on CT scans[3]. Since they are removed after the CT scan, they must be carefully excluded from the External contour by checking in the **Bone** (450/1600 HU) window-level setting. If forgotten, the External contour must be adjusted before the plan can be accepted.

S3A.6 Coated feeding tubes

Many esophagus cancer patients require tube feeding[4]; some patients have it from the first fraction, and some get it during the treatment course. The size of the tube ranges from 4 to 5 mm, and the plastic inner wall is about 1 to 2 mm thick. Some tubes have iodine coatings, which appear denser on CT scans, risking overestimation of their size when overridden. For example (Figure S9), in the **Mediastinum** (40/400 HU) window-level setting, the tube may appear about 7 mm in diameter, but in the **Bone** (450/1600 HU) setting, its hollow structure is visible. The iodine coating results in CT numbers above 1200 HU in some pixels. Based on our initial evaluations (see main text), we decided not to override feeding tubes.

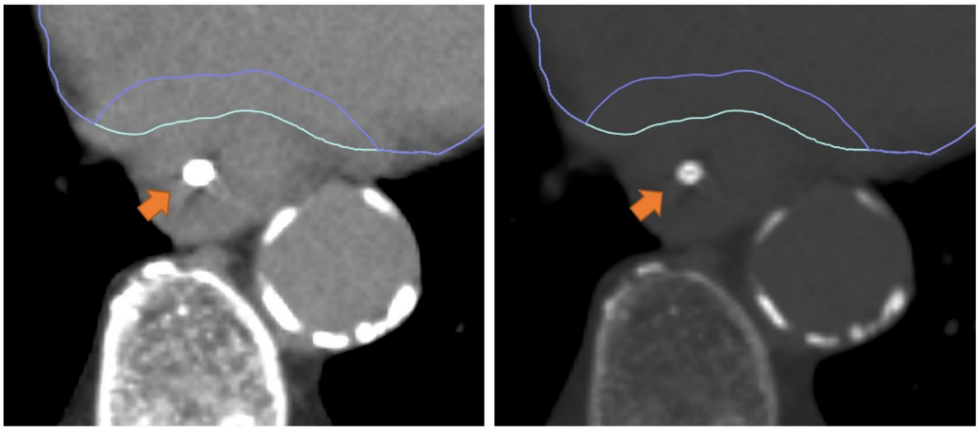

Figure S9: CT image of an esophagus patient with iodine coated feeding tube. (Left) Mediastinum window-level setting (40/400 HU); in this window-level setting, the size of the tube is overestimated (measured diameter 0.69 cm), and it cannot be seen that the tube is hollow. (Right) Bone window-level setting (450/1600 HU); here the dimension of the iodine coating is closer to reality (measured diameter 0.65 cm), and the hollow structure of the tube is visible.

S3B Large implants

As a general rule, "large" implants are always contoured and overridden to the corresponding material. Even if the implant is made of titanium which is included in the CT conversion curve, they need to be overridden because the CT numbers inside the material might not be uniform. Whenever possible, we do not shoot a proton beam through the implant, and we have a critical look at the streak artifacts to assess if the CT numbers in the surrounding area are reliable.

S3B.1 Breast implants

Breast implants or prostheses are made from a silicone rubber shell filled with either sterile saline solution (salt water) or silicone gel. The thickness of the shell is less than 1 mm[5].

S3B.1a Tissue expander

Breast reconstruction may be performed after mastectomy and, as part of the reconstruction process, a breast tissue expander may be used to stretch the patient's tissue for the insertion of an implant or the patient's own tissue at a later time[6,7].

Tissue expanders are like thick-walled silicone balloons, come in different sizes and shapes, and are filled with saline water. They have a metallic port or valve[6]. There are many different implant models, and the shape of the metallic port differs for each model (see e.g. Figure 1 in reference[6]). The type of valves that we have seen so far in our patients are similar to the model shown in Figure 1A in from Kang *et al.*[6] (Mentor CPX4; Mentor Worldwide, Irvine, CA).

We delineate the valve in the **Bone** window-level setting (450/1600 HU) and double check if the resulting contour's dimensions are similar to the real dimensions of the valve according to the manufacturer's specifications. Then, we override the valve contour to steel. When creating the proton plan in RayStation, we use an OAR range margin[*1] for each beam for the valve structure expanded by 3 to 5 mm (depending on the proximity with the target volume). This will ensure that no spots are placed on the metallic valve. In addition, to evaluate the influence on the dose of a potential displacement of the valve inside the breast volume, we

[*1] OAR range margin: Functionality in RayStation to control unwanted placement of proton spots. No spots will be placed within the specified contour, and if a margin larger than 0 cm is chosen, spots are also not placed outside the contour within this specified distance. If not otherwise specified in this document, this distance is set to 0 cm, so no spots are placed within the specified contour, but spots can be placed right outside the contour[8].

generate deformed CTs using the Simulate Organ Motion functionality in RayStation with a 3 or 5 mm displacement of the valve setup and then we re-compute the dose on these CTs.

S3B.1b Silicone implant

The implant is contoured with window-level setting **Spine** (35/300 HU), including the external silicone rubber. An example of a patient with a silicone implant is shown in Figure S10. Based on the analysis described in the main text, we found that the best strategy is to override silicone implants to water and then adjust the mass density to 0.934 g/cm^3, to reach a SPR value as measured in the study by Michalak *et al.*[9]

Special consideration: In case of a lung patient with implants and beams going through the implant, we try to avoid anterior oblique beams, when possible, to reduce the path length through the implants.

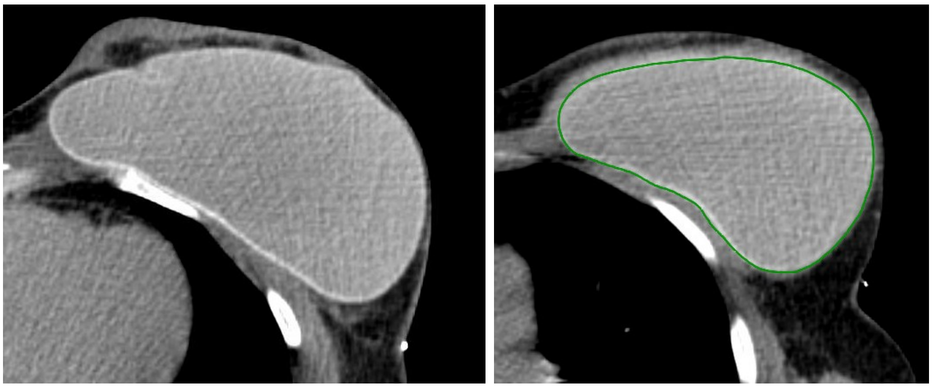

Figure S10: Example of a CT image of a silicone breast implant, where the silicone rubber shell is visible. In the right figure, the implant is contoured in green. Window-level setting Spine (35/300 HU).

Material override evaluation:

As material override directly to silicone (plastic material) is not possible in RayStation, we evaluated whether to perform a material override to silicon (pure material, Z=14) or water. In this evaluation, we had two criteria, namely the SPR (and thereby the proton dose) should be correctly simulated, and the proton scattering conditions should be reliably reproduced. A simplified setup was implemented in RayStation, in which a single proton spot was delivered through air, water, or silicon, to evaluate the material-specific interactions. The simulations in air were only for reference. In the simulations for water and silicon, their mass densities were adjusted to reproduce the SPR of silicone. For all three materials, simulations with proton energies of 155, 74.4, 50.6, 25 MeV were performed. Two different geometries were used; for 155 and 74.4 MeV, a box with a 3 cm thickness in the beam direction was used, and for 25 and 50.6 MeV, the box had a thickness of 0.5 cm in the beam direction (to ensure that the beam penetrated the box). In the beam direction, this box was followed by water with an unadjusted mass density. The proton beam would for all four energies stop in the regular water, whereby the beam range could be measured, by integrating (summing) the dose perpendicular to the beam directions in steps of 1 mm along the beam direction. The beam range was obtained by interpolation of the deposited dose on the distal part of the Bragg peak.

For the water material, the mass density was set to 0.934 g/cm^3 to give a SPR of 0.934 corresponding the SPR measured by Michalak *et al.*[9] For silicon, the SPR, and thereby the mass density, would be slightly dependent on the proton energy. For a proton energy of 155 MeV, we calculated the mass density to 1.158 g/cm^3, when using a mean excitation energy of 75 eV for water and of 173 eV for silicon. Similar calculations for energies 74.4 MeV, 50.6 MeV, and 25 MeV resulted in densities for silicon of 1.1691 g/cm^3, 1.1764 g/cm^3, and 1.1923 g/cm^3, respectively. This was already a good indicator that using silicon as the override material is not viable because it requires an energy-dependent mass density override.

The extracted depth-dose curves for the adjusted water and adjusted silicon material were exactly the same, showing that with the correct mass density adjustment, the proton range would be similar independent of the

chosen override material. In addition, the SPR determined from the measured proton range was in all eight tests (four energies and two materials) in close agreement with the measured SPR of 0.934. The best agreement was found for the 155 MeV beam (deviations of ~0.3%). Larger deviations were found for the 50.6 MeV and especially 25 MeV beam, but for these, the lateral scattering (i.e. the proton spot size) was larger and they only passed through 0.5 cm of the adjusted water or silicon material. We therefore concluded that both override materials simulated silicone well.

Extracting lateral line profiles, the scattering of the proton beam after passing through adjusted water (effective atomic number of ~7.5) and through adjusted silicon (atomic number of 14) was almost identical. It was therefore concluded that it was okay to use water as base material for material override, and then adjust the mass density to 0.934 g/cm^3 to give the correct SPR[9].

S3B.1c Saline implant

An example of a patient with a saline implant is shown in Figure S11. The CT numbers for the saline implant range from -20 to 20 HU, i.e., close to water. The saline implant does not need to be overridden (salt water is correctly estimated using the CT conversion curve). The silicone rubber shell has CT numbers ~200 HU, and in some CT slices it can look like it has a thickness of ~3 mm if the incorrect window-level setting is chosen, but the real dimension of the rubber wall is ~1 mm. By not overriding the silicone rubber wall, we introduce dose differences in some dose grid voxels because the CT number of the rubber is ~200 HU, so the SPR estimated based on the CT conversion curve would be ~1.2, but the SPR of silicone is 0.934[9]. However, the error that we would introduce by contouring wrongly (too thick) the rubber wall might be higher. Therefore, we have chosen not to contour nor override the rubber wall. Therefore, in case of saline implants, we do not contour and override anything.

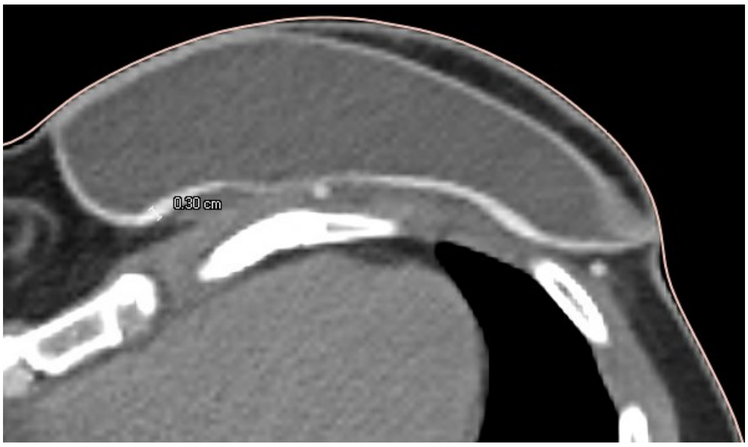

Figure S11: Example of a CT image of a patient with a saline breast implant. The silicone rubber surrounding shows up light on the CT image due to the high CT numbers (~200 HU). Window-level setting Mediastinum (40/400 HU).

S3B.2 Dental fillings

Dental fillings are often made of metals or ceramics[10]. We always reconstructed CT scans of head-and-neck cancer patients using iMAR with the setting “dental filling”. To contour the fillings, we adjust the window-level settings so there is a clear distinction between the teeth and the fillings. When creating the treatment plan, the proton beam should never go through the dental fillings. When creating the plan in RayStation, an OAR range margin is used for the dental filling contour, so no spots are placed within the filling. We do not override dental fillings to any material because the current clinical practice is not to go through it with the proton beam.

S3B.3 Totally implantable venous access devices

An implantable port (also called Portacath or Chemo port) is a small central venous access port that is placed under the skin of the right-side chest area (Figure S12). The port is connected to a catheter which is inserted

into a large vein and threaded into a place near the heart. Patients that require frequent administration of medications and/or fluids directly into the blood vessel receive this implant, this includes patients receiving chemotherapy. It is usually made of surgical steel or titanium, and it is around 2.5 to 4 cm in diameter. The head of the port has a rubbery membrane made of silicone[11].

The proton beam should not go through the port. In case of a left-side breast cancer patient with a Portacath, we apply an OAR range margin, with an additional margin equal to the setup uncertainty, for the port contour. The implant can be contoured in RayStation by using window-level setting **Pelvis** (250/1000 HU) and s*mart brush* tool (radius ~0.45 cm, bright edge detection, 85% sensitivity). We do not override implantable venous access devices to any material because the current clinical practice is not to go through it with the proton beam.

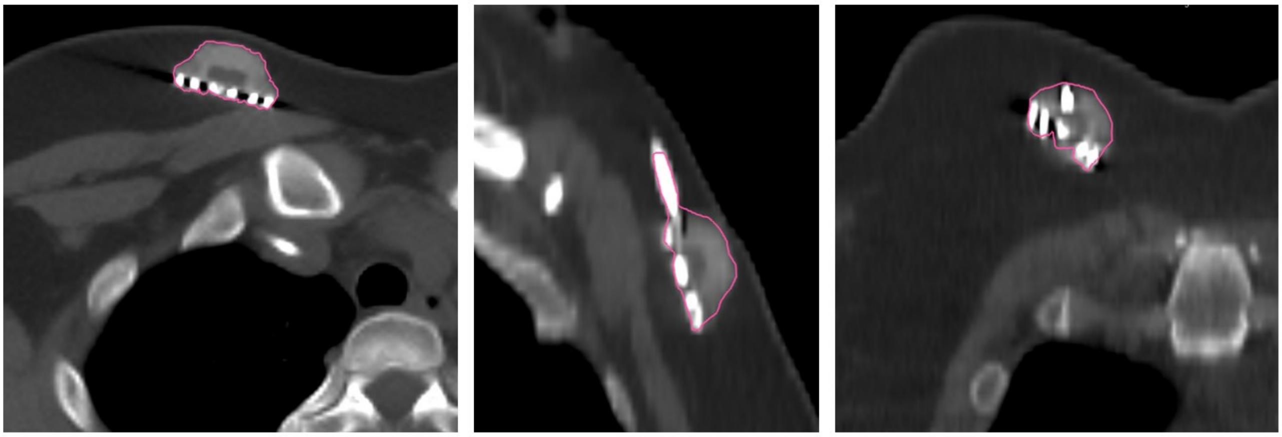

Figure S12: CT image of a patient with a Portacath (pink contour) in transversal, sagittal and coronal views. Window-level setting Pelvis (250/1000 HU).

S3B.4 Pacemakers wires

We reconstruct CT scans of patients with a pacemaker with iMAR using the "neuro coils" setting. Careful beam adjustment is required to avoid the pacemaker in the proton beam path. Additionally, we use an OAR range margin with a margin equal to the setup uncertainty to prevent direct proton beam exposure to the pacemaker. The pacemaker wire is typically made of a NiCoCrMo alloy with a density of ~8.4 g/cc[12]. The wire may be near the target volume, and if a proton beam must pass through it, we contour the wire and override it to steel, as steel is the closest match in RayStation 12A to NiCoCrMo. However, this remains an approximation and may cause some dose uncertainty that physicians should be aware of. To assess the influence of this incorrect material override, we evaluated the effect of overriding the wire to titanium (mass density 4.5 g/cm^3) as well as not overriding it for the patient shown in Figure S13. For this patient, there were no differences in target and OAR DVH parameters as the wire is only in the distal part of the beams.

For thoracic tumor patients (esophagus, lung, lymphoma, thymoma), we acquire a 4DCT scan for the treatment planning and dose recalculation. The delineation is performed on the 50% expiration phase (CT50ex) and the dose calculation is performed on the average CT[13]. However, implants are contoured directly on the average CT, using the **Bone** (450/1600 HU) setting, especially if the implant is near the heart or diaphragm, since motion may then affect the contour, leading to inaccuracies. Figure 4 in the main text shows how motion can result in misaligned contours when copied from CT50ex to average CT, leading to an unnecessary override of voxels inside the target volume.

We avoid using a beam configuration that passes through the pacemaker wire due to the lack of an equivalent material in RayStation 12A. In cases like the one shown in Figure S14, where the pacemaker lead is close to the target volume, using an OAR margin with an additional margin would result in severe under-dosage. Instead, we contour the wire with the **Bone** (450/1600 HU) setting using the *smart brush* tool (radius 0.5 cm or smaller, bright edge detection, 80% sensitivity), and override it to steel. To prevent over-contouring, only the brighter part of the wire is contoured, and the artifacts are not overridden.

In Figure S15 there is another example of a lymphoma patient with a pacemaker wire that overlaps with the target volume. The wire was contoured following the above description, but the OAR range margin was avoided because this was a case of salvage radiotherapy, and no target concessions could be made. The general consideration is therefore, for patients for whom there is overlap between the wire and the target, the unavoidable dose uncertainty should be weighed against the gain of receiving proton therapy, and as in the case shown in Figure S15, the compromise might be to accept a higher uncertainty. In this particular case, the patient received photon therapy because of the limited gain in normal tissue complication probability[14].

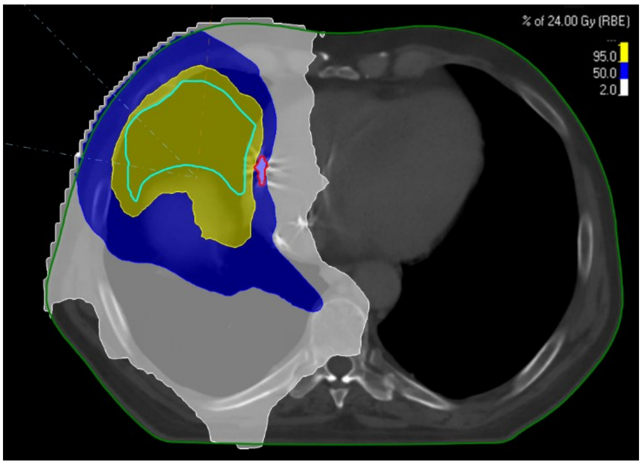

Figure S13: Proton dose distribution for patient with pacemaker wire (red contour) close to the target (cyan contour). None of the three beams (dashed lines in upper left corner of figure) pass the wire before reaching the target.

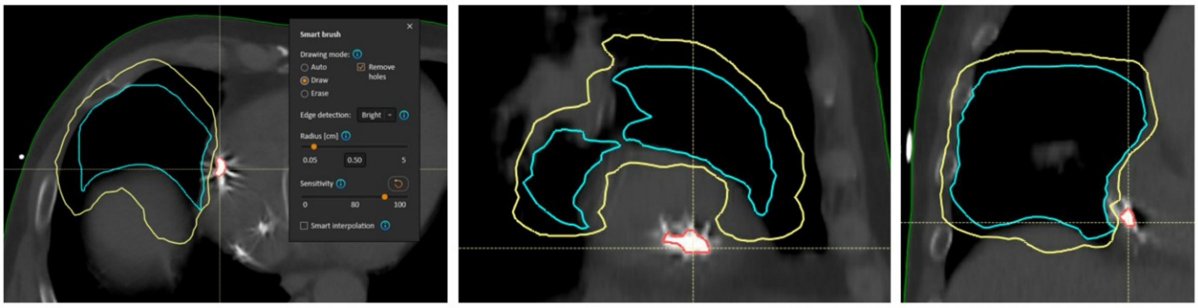

Figure S14: Example of a CT image of a patient with a pacemaker wire (red contour) right outside the target volume (blue contour: clinical target volume (CTV); yellow contour: 95% iso-dose line). The contouring of the wire is done with the smart brush (left figure). Window-level setting Bone (450/1600 HU).

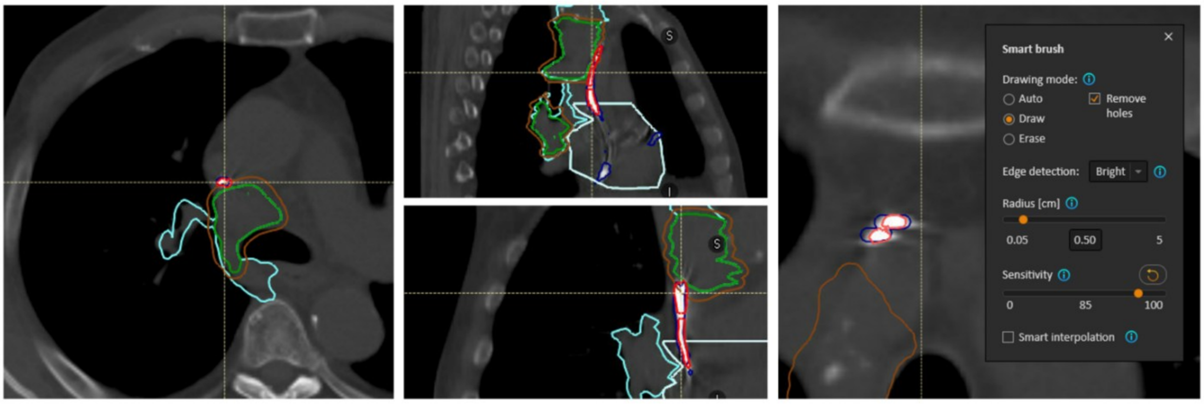

Figure S15: Lymphoma cancer patient with a pacemaker wire (red contour) overlapping with the target (dark green contour: CTV2; brown contour: CTV2 expanded by 3 mm (target)). The red contour is created directly on the average CT, applying the smart brush tool (right), while the dark blue contour is created on the CT50ex. Window-level setting Bone (450/1600 HU).

S3B.5 Coronary stents

A stent is a short, narrow tube made of a metal or plastic mesh, inserted into vessels to keep them open. Coronary artery stents are typically made of metal mesh, while fabric stents (stent grafts) are used in larger arteries like the aorta. Common metals used for stents include stainless steel, and alloys of cobalt-chromium, platinum, tantalum, and niobium[15].

Despite being made of metal mesh, coronary artery stents often appear as large, blurred structures on average CT scans, with CT numbers ranging from ~60-660 HU and an average of around 340 HU (Figure S16). The metal mesh is around 95% air and 5% metal. By relying on the CT conversion curve, for a structure with a mean CT number of 340 HU, the estimated SPR would be ~1.3. Given the uncertainties in choosing the most appropriate material to override to, we assume that the error we would make by not overriding is smaller than the error that we would commit by over overriding it wrongly, so no material override is performed. In Figure S17, the stent was contoured with window-level setting **Soft Tissue** (40/350 HU) to measure the mean CT number, but no material override was performed.

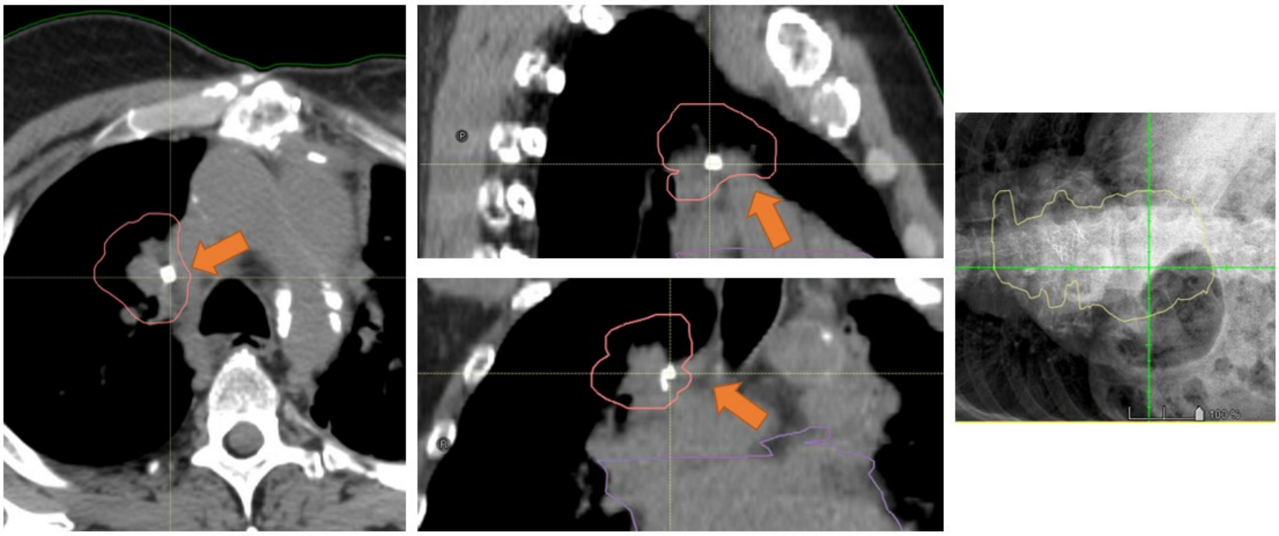

Figure S16: Example of a patient with a coronary artery stent. The left and middle figures are an average CT scan (the orange arrows points to the stent). The stent looks much bigger and solid on the CT image, while on the X-ray on the right, it is clearly seen that the stent is a mesh and not a solid metal object.

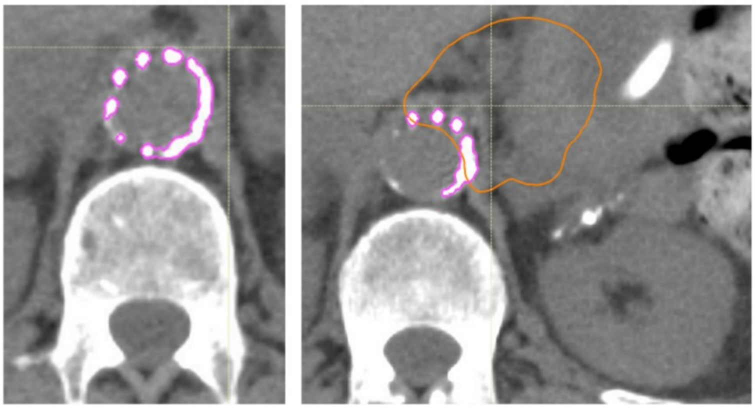

Figure S17: Example of a patient with an aorta stent (purple contour). The figure shows two different CT slices. The CT numbers for the stent contour range from ~23-2437 HU, with an average of 441 HU. Window-level setting Soft Tissue (40/350 HU).

S3B.5 Air pockets in the esophagus

As described in the main text and shown in Figure S18, the mean dose to the heart increases with the increase in the air pocket size – note, we generally use posterior beams. Based on these results and our clinical experience, we do not contour air pockets smaller than 15 mm in diameter. For air pockets larger than 15 mm,

we contour and override them to water with a reduced density of 0.5 g/cm³. We then evaluate the plan recalculated on a copy of the CT without any override[16]. We do not evaluate air pockets in organs-at-risk like the stomach or trachea.

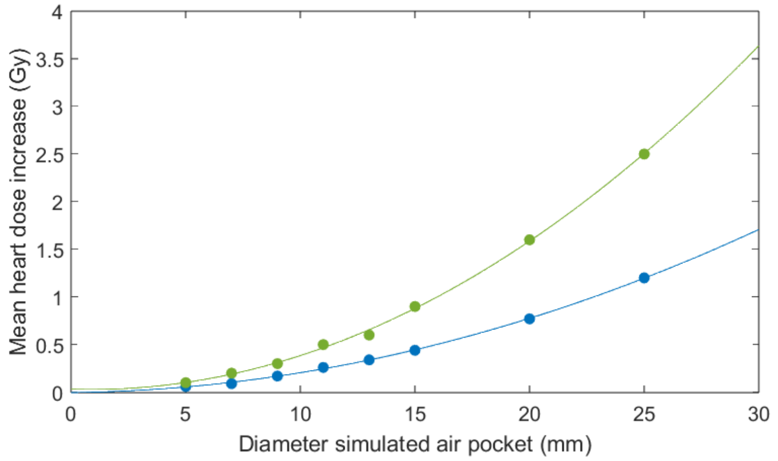

Figure S18: Increase in mean heart dose as a function of the diameter of the simulated air pocket. The lines are second order polynomial fits to the data.

S3B.6 Gastric band

The laparoscopic adjustable gastric band is a surgically implanted device to help with weight loss by restricting food intake. It is an inflatable silicone band placed around the upper stomach, forming a ring that reduces stomach size without permanently dividing it. The band, typically 13 mm wide, comes in two sizes (9.75 cm and 10 cm inner circumference) and is connected to a stainless steel port under the skin, allowing adjustments via saline injection[17]. A 50 cm silicone tube links the band to the port, which can shift within the peritoneum. Scatter artifacts from the metallic port can be seen in CT scans (Figure 1 in reference[18]). The band (wall thickness ≤1 mm) and the connector tube are made of silicone and they are hollow[19].

If the band or tube is near the target volume (Figure S19), we duplicate the CT scan and plan on the original CT without material override. On the copied CT, we contour the tube using the **Spine** (35/300 HU) window-level setting, applying the smart brush tool (radius 0.3 cm, bright edge detection, 80% sensitivity). This contour is overridden to silicone (water as basis and mass density 0.934 g/cm³). The plan is then re-computed and robustly evaluated on the CT with material override. Not overriding the silicone tube introduces dose differences due to its CT number (~170 HU), resulting in an SPR of ~1.1, while silicone has an SPR of 0.934. However, since the tube can move within the target, we prefer to plan on the CT without material override and only evaluate the plan robustness on the CT with the override.

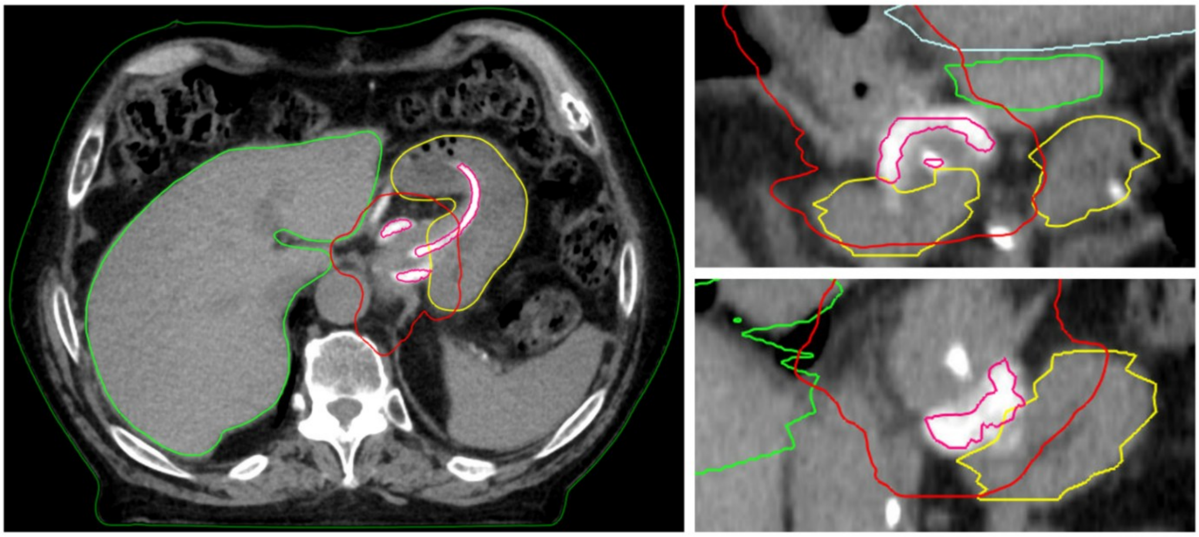

Figure S19: Example of an esophagus cancer patient with a gastric band (pink contour; red contour: target volume; yellow contour: stomach; green: liver; light blue: heart. Left: transversal; right upper: sagittal; right lower: coronal view. The gastric band is contoured following the proposed procedure. The CT numbers range from 15 HU to 317 HU, with an average of 167 HU. Window-level setting Spine (35/300 HU).

S3B.7 Hip, shoulder, spine metal implant

In some cases, we do treat patients with large implants inside or around the target volume with proton therapy (Figure S20). However, we only treat these patients with protons if the implant is accurately delineated, and the implant material is well known. In such cases, shooting through the implant may be unavoidable and using multiple proton beam angles may be necessary to ensure proper target coverage. However, if the implant material is unknown or not listed in RayStation, proton therapy cannot be safely administered, and the patient cannot be treated with protons in our clinic. An exception is made if the SPR of the implant is well established. In this case, a new material can be created in RayStation, using water as a base and adjusting the mass density to match the implant material's SPR (see example for silicone in the main text).

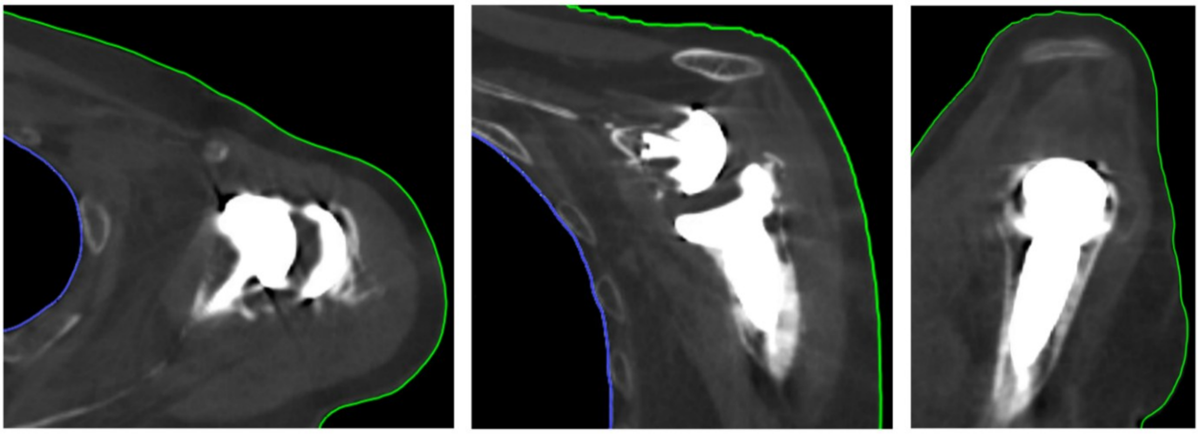

Figure S20: Shoulder implant in the left arm of a patient who was treated for a lung tumor in the right lower lobe. It was therefore possible to treat the patient without having any proton beams shoot through the implant. Window-level setting Bone (450/1600 HU).

S4 References

1. Peters N, Taasti VT, Ackermann B, et al. Consensus guide on CT-based prediction of stopping-power ratio using a Hounsfield look-up table for proton therapy. Radiother Oncol. 2023;184:109675. doi:10.1016/j.radonc.2023.109675

2. Tsunezuka Y, Tanaka N, Fujimori H. The Impact of Endoscopic Stapler Selection on Bleeding at the Vascular Stump in Pulmonary Artery Transection. Med Devices: Evid Res. 2020;Volume 13:41-47. doi:10.2147/MDER.S240343

3. Hurkmans CW, Borger JH, Pieters BR, Russell NS, Jansen EPM, Mijnheer BJ. Variability in target volume delineation on CT scans of the breast. Int J Radiat Oncol Biol Phys. 2001;50(5):1366-1372. doi:10.1016/S0360-3016(01)01635-2

4. Li Y, Pond G, Van Osch A, et al. Enhancing Nutrition Support for Esophageal Cancer Patients: Understanding Factors Influencing Feeding Tube Utilization. Nutr Cancer. 2024;76(3):271-278. doi:10.1080/01635581.2024.2301796

5. Jewell ML, Bengtson BP, Smither K, Nuti G, Perry T. Physical Properties of Silicone Gel Breast Implants. Aesthet Surg J. 2019;39(3):264-275. doi:10.1093/asj/sjy103

6. Kang Y, Shen J, Bues M, Hu Y, Liu W, Ding X. Technical Note: Clinical modeling and validation of breast tissue expander metallic ports in a commercial treatment planning system for proton therapy. Med Phys. 2021;48(11):7512-7525. doi:10.1002/mp.15225

7. Yoon J, Xie Y, Heins D, Zhang R. Modeling of the metallic port in breast tissue expanders for photon radiotherapy. J Applied Clin Med Phys. 2018;19(3):205-214. doi:10.1002/acm2.12320

8. Janson M, Glimelius L, Fredriksson A, Traneus E, Engwall E. Treatment planning of scanned proton beams in RayStation. Med Dosim. 2024;49(1):2-12. doi:10.1016/j.meddos.2023.10.009

9. Michalak G, Taasti V, Krauss B, Deisher A, Halaweish A, McCollough C. A comparison of relative proton stopping power measurements across patient size using dual- and single-energy CT. Acta Oncol. 2017;56(11):1465-1471. doi:10.1080/0284186X.2017.1372625

10. Wong YM, Koh CWY, Lew KS, et al. Effects of modern aesthetic dental fillings on proton therapy. Phys Imaging Radiat Oncol. 2024;29:100552. doi:10.1016/j.phro.2024.100552

11. Niederhuber JE. Totally Implantable Venous Access Devices. (Di Carlo I, Biffi R, eds.). Springer Milan; 2012. doi:10.1007/978-88-470-2373-4

12. Padera RF, Schoen FJ. Cardiovascular Medical Devices. In: Biomaterials Science. Elsevier; 2020:999-1032. doi:10.1016/B978-0-12-816137-1.00067-2

13. Taasti VT, Hattu D, Vaassen F, et al. Treatment planning and 4D robust evaluation strategy for proton therapy of lung tumors with large motion amplitude. Med Phys. 2021;48(8):4425-4437. doi:10.1002/mp.15067

14. Vishnu J, Manivasagam G, Mantovani D, et al. Balloon expandable coronary stent materials: a systematic review focused on clinical success. In vitro models. 2022;1(2):151-175. doi:10.1007/s44164-022-00009-w

15. Loewe C, Diaz F, Jackson A. LAP-Banding Obesity: A Case of Stomach Perforation, Peritonitis, and Death. Am J Forensic Med Pathol. 2005;26(3):297-301. doi:10.1097/01.paf.0000176282.71566.69

16. Tan LB, So JB, Shabbir A. Connection tubing causing small bowel obstruction and colonic erosion as a rare complication after laparoscopic gastric banding: a case report. J Med Case Reports. 2012;6(1):9. doi:10.1186/1752-1947-6-9

17. Furbetta N, Cervelli R, Furbetta F. Laparoscopic adjustable gastric banding, the past, the present and the future. Ann Transl Med. 2020;8(S1):S4-S4. doi:10.21037/atm.2019.09.17
